# Inflammasomes, Autophagy, and Cell Death: The Trinity of Innate Host Defense against Intracellular Bacteria

**DOI:** 10.1155/2019/2471215

**Published:** 2019-01-08

**Authors:** Teresa Krakauer

**Affiliations:** Department of Immunology, Molecular and Translational Sciences Division, United States Army Medical Research Institute of Infectious Diseases, Fort Detrick, Frederick, MD 21702-5011, USA

## Abstract

Inflammasome activation is an innate host defense mechanism initiated upon sensing pathogens or danger in the cytosol. Both autophagy and cell death are cell autonomous processes important in development, as well as in host defense against intracellular bacteria. Inflammasome, autophagy, and cell death pathways can be activated by pathogens, pathogen-associated molecular patterns (PAMPs), cell stress, and host-derived damage-associated molecular patterns (DAMPs). Phagocytosis and toll-like receptor (TLR) signaling induce reactive oxygen species (ROS), type I IFN, NF*κ*B activation of proinflammatory cytokines, and the mitogen-activated protein kinase cascade. ROS and IFN*γ* are also prominent inducers of autophagy. Pathogens, PAMPs, and DAMPs activate TLRs and intracellular inflammasomes, inducing apoptotic and inflammatory caspases in a context-dependent manner to promote various forms of cell death to eliminate pathogens. Common downstream signaling molecules of inflammasomes, autophagy, and cell death pathways interact to initiate appropriate measures against pathogens and determine host survival as well as pathological consequences of infection. The integration of inflammasome activation, autophagy, and cell death is central to pathogen clearance. Various pathogens produce virulence factors to control inflammasomes, subvert autophagy, and modulate host cell death in order to evade host defense. This review highlights the interaction of inflammasomes, autophagy, and host cell death pathways in counteracting *Burkholderia pseudomallei*, the causative agent of melioidosis. Contrasting evasion strategies used by *B*. *pseudomallei*, *Mycobacterium tuberculosis*, and *Legionella pneumophila* to avoid and dampen these innate immune responses will be discussed.

## 1. Introduction

Neutrophils and macrophages are professional phagocytes that act together in innate host defense against invading bacteria [1–3]. These cells capture, phagocytose, and engulf bacteria into phagosomes. Phagosome maturation by sequential fusion with endocytic and lysosomal compartments results in the formation of an acidic and oxidative phagolysosome to degrade microbes. Macrophages are long-lived cells, and intracellular entry into these cells allows bacteria to persist and spread to other organs and tissues. Pathogenic microbes manipulate host macrophages to reside and replicate in these cells. Macrophages provide a nutrient-rich environment and shelter microbes from extracellular host defense such as complement-mediated lysis or uptake by neutrophils for degradation [4, 5]. Neutrophils have a shorter life span and higher microbicidal activities compared to macrophages, releasing degradative granule proteases and antimicrobial peptides upon activation. At sites of infection, activated macrophages release cytokines and chemokines to recruit neutrophils to infected areas. Once inside the macrophage, intracellular bacteria can live in vacuolar compartments or the cytosol, depending on their virulence factors which help them to evade host defense and replicate in these compartments [5]. Macrophages act as professional antigen-presenting cells in the development of adaptive immune response to pathogens. In addition, macrophages are also responsible for tissue homeostasis by phagocytosis of apoptotic cells and facilitate tissue repair and resolution of inflammation.

In the past few years, the biodefense community has undertaken research on the facultative, gram-negative bacterium, *B. pseudomallei*, because it is highly pathogenic, and melioidosis is difficult to treat contributing to high mortality in endemic regions [6, 7]. Infection with *B. pseudomallei* results in various outcomes, ranging from acute fatal sepsis to chronic infection with or without clinical symptoms [6]. Prolonged combinational antibiotic therapy is required for treatment and to prevent relapse of melioidosis [7]. The high rate of infection recurrence and the possibility of latent infection with *B. pseudomallei* indicate the bacterium evades host defense mechanisms successfully. *B. pseudomallei* is resistant to the early microbicidal activities of macrophages and neutrophils after phagocytosis and replicates in both cell types *in vitro* [8]. *B. pseudomallei* and its virulence factors counteract and/or evade innate host defense components in phagocytes early to persist intracellularly. This review highlights the cross-regulation of three innate components of host defense: inflammasomes, autophagy, and cell death in counteracting the intracellular bacterium *B. pseudomallei*. The evasion strategies used by pathogenic bacteria such as *Mycobacterium tuberculosis* (*Mtb*), *Legionella pneumophila*, and *B. pseudomallei* to subvert these innate cellular processes will also be discussed.

## 2. Host Defender Macrophage as an Intracellular Niche for Bacteria

Macrophage phagocytosis of microbes activates NADPH oxidase resulting in reactive oxygen species (ROS) and inducible nitric oxide synthase which generates nitric oxide (NO). ROS and NO are antimicrobial and mediate the killing of microbes upon their engulfment in phagosomes [1]. Innate mediators, type I IFN (IFNI includes IFN*α* and IFN*β*) and type II IFN (IFN*γ*), are potent activators of NO and ROS [9, 10]. Both types of IFNs also induce the expression of macrophage NADPH oxidase subunits to enhance ROS production [10]. Many intracellular bacteria including *B. pseudomallei* avoid colocalization with ROS and NO by escaping the phagosome. Bacteria use type III, type IV, type VI, or type VII secretion systems to inject diverse effectors to evade these potent antimicrobial molecules and escape various phagosomal compartments inside the macrophage [11, 12]. For example, the type III secretion system (T3SS) effectors of *B. pseudomallei* aid bacterial escape from the phagosome into the cytosol of mouse macrophages, where it replicates [13, 14]. In addition, the extracellular polysaccharide capsule of *B. pseudomallei* reduces macrophage phagocytosis by decreasing complement deposition [15]. The T4SS Icm/Dot apparatus of *L. pneumophila* inhibits phagosomal fusion with lysosomes and recruits endoplasmic reticulum (ER) vesicles to form a specialized vacuole for *Legionella* replication [16]. *Mtb* secretes SapM, a lipid phosphatase that hydrolyzes phosphatidylinositol 3-phosphate to prevent phagosome maturation and survive within the phagosome [17]. Pore-forming toxin, listeriolysin O, mediates the escape of *Listeria monocytogenes* from the phagolysosome and specialized vacuoles into the cytosol [18, 19]. *Francisella tularensis* uses T6SS effectors to escape endosome-like vacuoles and phagosomes to replicate in the cytosol [20]. Thus, pathogenic bacteria have evolved different mechanisms to subvert initial macrophage antimicrobial defense and replicate within these phagocytes [4, 5].

## 3. Autophagy in Host Defense against Intracellular Bacteria

Bacteria that escape the phagosome and phagosomal bacteria can be targeted for elimination by another innate component of host defense termed xenophagy, a selective form of autophagy [21–25]. Autophagy is an evolutionarily conserved cellular process for sequestration of cytoplasmic protein aggregates, dysfunctional organelles, and microbes for lysosomal degradation [21, 22]. Successful pathogens subvert the autophagic process or evade autophagy to avoid degradation [21–25]. Autophagy is activated by phagocytosis and pattern recognition receptors (PRRs), especially toll-like receptor 4 (TLR4) sensing of bacterial lipopolysaccharide (LPS), and is dependent on ROS generation from phagocytes [26]. In canonical autophagy, phagosomal intracellular microbes are enclosed by a double-membrane autophagosome which then fuses with lysosomes to form an autolysosome. Initiation of canonical autophagy requires ULK1 (Unc-51-like kinase 1), beclin 1, microtubule-associated protein light chain 3 (LC3), phosphatidylinositol 3-kinases, and many autophagy-related proteins (Atgs). LC3 conjugation onto a phagosome promotes the fusion of phagosome with lysosome. The capture of intracellular *B*. *pseudomallei* relies on a noncanonical autophagic process called LC3-associated phagocytosis (LAP). LAP uses a single membrane phagosome to enclose bacteria, which then fuses with lysosomes for degradation of bacteria [21–25]. Briefly, LAP requires a fewer number of Atg proteins and is highly dependent on NADPH oxidase 2 and phosphatidylinositol 3-phosphate for LC3 conjugation onto a phagosome [23, 26]. The T3SS effector protein BopA facilitates the escape of *B*. *pseudomallei* from LAP in mouse Raw264.7 cells [27]. BipD, a T3SS translocator protein, also participates in phagosomal escape of *B*. *pseudomallei* into the cytosol [28]. Importantly, evasion of autophagy improves *B*. *pseudomallei* survival in mouse macrophages. This is consistent with previous observations that most *B*. *pseudomallei* escape phagosomal degradation in human macrophages and neutrophils and replicate in the cytosol of these phagocytes [8, 29].

*Mtb*, the causative agent for tuberculosis, uses multiple mechanisms to subvert autophagy [30]. *Mtb* limits autophagy by preventing phagosomal maturation [31]. *Mtb* also blocks autophagosomal maturation, by inhibition of phagosome acidification. In contrast, *L*. *pneumophila*, an opportunistic human pathogen, inhibits autophagy directly by secretion of RavZ, a cysteine protease which cleaves phosphatidylethanolamine (PE) from PE-conjugated LC3 and inhibits autophagosome formation [32].

## 4. Cooperative Sensing of Bacteria by Pattern Recognition Receptors

Macrophages sense pathogens by using pattern recognition receptors (PRRs) to detect pathogen-associated molecular patterns (PAMPs) and host-derived damage-associated molecular patterns (DAMPs) induced by microbes and cell damage [33–37]. Cell surface PRRs, which include toll-like receptors (TLRs) and C-type lectin receptors (CLRs), detect bacteria and their secreted products extracellularly. Endosomal TLR3, TLR7, TLR8, and TLR9 sense bacterial and viral nucleic acids, inducing IFNI and proinflammatory cytokines [34–36]. Cytosolic PRRs include nucleotide-binding domain and leucine-rich repeat-containing protein (NLR) family members, absent in melanoma 2- (AIM2-) like receptors (ALRs), retinoic acid-inducible gene I- (RIG-I-) like receptors (RLRs), pyrin and HIN domain-containing family members, and other cytosolic nucleic sensors [38].

### 4.1. Toll-Like Receptors and Bacterial Ligands

Extracellular TLR2 and TLR4 detect bacterial lipoproteins and lipopolysaccharide (LPS), respectively, to activate intracellular myeloid differentiation protein 88- (MyD88-) dependent NF*κ*B and induce proinflammatory cytokines including IL1*β* and TNF*α*. An additional MyD88-independent TIR-domain-containing adaptor protein-inducing interferon-*β* (TRIF) pathway is triggered by TLR3 and TLR4 upon interaction with their respective ligands, dsRNA and LPS, to induce IFN*β* via TRIF phosphorylation of the transcription factor IFN regulatory factor 3. Cell stress response pathways such as the mitogen-activated protein kinase (MAPK) cascade are also upregulated by various TLRs via MyD88. IL1*β* and TNF*α* are generally host protective. IL1*β* promotes neutrophil recruitment for pathogen clearance whereas TNF*α* stimulates IFN*γ* production from natural killer cells and T cells, initiates apoptosis, and activates MAPKs and the production of other cytokines [39]. IFNI has variable cell-type-specific effects and enhances host susceptibility to intracellular bacteria [40]. Tonic IFNI signaling protects against pathogens early in an infection, but sustained IFNI is detrimental during bacterial infection [41]. Some pathogenic bacteria produce virulence factors to downregulate TLR and cytokine signaling. For example, *B*. *pseudomallei* secretes a deubiquitinase TssM inside the macrophage to interrupt TLR-mediated NF*κ*B signaling and proinflammatory cytokine induction [42]. TssM mutants are less virulent in mice and exhibit higher inflammation. *Yersinia pestis* virulence factor YopJ inhibits NF*κ*B and MAPK signaling to dampen inflammation [43]. Virulent *Mtb* strains induce IFNI to limit IL1*β* production [44]. *Mtb* also inhibits IFNI signaling to promote its pathogenicity [45].

LPS, a conserved PAMP present in the outer membranes of gram-negative bacteria, is a potent activator of TLR4 [46]. LPS binds to host LPS-binding protein (LBP) and CD14 which then recruit MD2 and TLR4 on the macrophage cell surface for intracellular signal transduction [47, 48]. LPS consists of a lipid A moiety conjugated to an O-linked polysaccharide. Lipid A, the active moiety of LPS, is usually hexa-acylated, and changes in acylation decrease its ability to activate TLR4 [49]. Pathogenic bacteria modify their cell wall components to avoid detection by TLRs [50]. *Y*. *pestis* deacylates lipid A from its hexa- to tetra-acylated forms at human body temperature to avoid detection by human TLR4 as the bacteria transition from the flea to the human host [51, 52]. *F*. *tularensis* also modifies the acylation of lipid A in response to temperature in order to maintain virulence in its host [53]. The tetra-acylated LPS of *B*. *pseudomallei* is a weak inducer of innate responses *in vitro* and *in vivo* [54]. Recently, hydroxylation was discovered as another modification that renders lipid A of *B*. *pseudomallei* less recognizable by TLR4 [55]. Various strains of *B*. *pseudomallei* with hydroxylated or tetra-acylated lipid A were found to be less cytotoxic and induce less TNF*α* in the mouse Raw264.7 cell line.

The cell wall components of *Mtb* are different from other intracellular bacteria. Cell wall lipomannan, lipoarbinomannan, lipoproteins, and mycolic acid of *Mtb* are mainly detected by TLR2 and, to a lesser degree, TLR4 [56]. TLR2 signaling activates NF*κ*B and MAPK cascades. TLR2-dependent recognition of *Legionella* peptidoglycan and lipopeptides has been shown to stimulate a protective response in mice that limits *Legionella* replication [57, 58]. The cytosolic NOD2 (nucleotide-binding domain and leucine-rich repeat-containing receptor 2) binds to the *Mtb* cell wall component peptidoglycan and induces IFNI [59].

In addition to TLR4, recent studies reveal activation of a cytosolic noncanonical inflammasome by direct binding of LPS to mouse caspase 11 or human caspase 4 and caspase 5 [60]. Importantly, caspase 11 is required for LPS-induced endotoxemia, whereas TLR4 only primes this response [61, 62]. Mouse caspase 11 also detects *B*. *pseudomallei* LPS and induces pyroptosis in macrophages and low levels of IL1*β* and IL18 by indirect activation of NLRP3 (NLR family pyrin domain-containing protein 3) [62]. TLR5 binds to flagellin and stimulates host response caused by flagellated bacteria, including *B*. *pseudomallei* and *L*. *pneumophila* [63, 64]. Flagellin is also detected by cytosolic sensors NLRC4 (NLR family CARD-containing protein 4) and NAIPs (NLR family apoptosis inhibitory proteins), which also detect various bacterial T3SS effectors [65].

### 4.2. Cytosolic Pattern Recognition Receptors Activate Inflammasomes

Intracellular PRRs patrol cytosolic compartments for pathogens, PAMPs, and DAMPs with NLRs, which serve as platforms for the assembly of multiprotein complexes called inflammasomes [66–69]. NLRs are classified based on their protein structure with the sensor and signaling domain-defining proteins that each NLR interacts with [70]. Most NLRs contain a C-terminal leucine-rich repeat domain which mediates ligand binding, a nucleotide-binding domain, and a N-terminal signaling domain which can be a pyrin domain or a caspase activation and recruitment domain (CARD) [70]. The signaling domain CARD recruits caspase 1 directly whereas pyrin domain receptors (NLRP3 and AIM2) require an additional adaptor ASC (apoptosis-associated speck-like protein containing a carboxy-terminal CARD) for interaction with caspase 1. NLRP3 and AIM2 oligomerize ASC through the interaction between the pyrin domain of ASC and the pyrin domain of NLR. The CARD of ASC then interacts with the CARD of caspase 1, resulting in autoproteolytic activation of caspase 1. Active caspase 1 then cleaves pro-IL1*β* and pro-IL18 and induces the extracellular release of active IL1*β* and IL18 and pyroptosis [71]. IL1*β* and IL18 have host protective functions as IL-1 recruits neutrophils to eliminate bacteria and IL18 induces IFN*γ* to activate phagocytes.

The triggers for activation of NLRs are different, with NLRP3 being the most complex, as it is activated by a diverse group of stimuli, including bacteria, viruses, fungi, pore-forming toxins hemolysin and nigericin, ATP, uric acid crystals, and peptide aggregates. Other proposed upstream activators of NLRP3 are ROS, mitochondrial DNA, phagosomal or lysosomal rupture, and potassium efflux [72]. Since these stimuli do not have a common structure, it is likely that induced cellular perturbation activates NLRP3. In contrast, NLRC4 and AIM2 inflammasomes recognize defined PAMPs. Bacterial T3SS effectors and flagellin activate NLRC4 after recruiting NAIP coreceptors without ASC [73, 74]. AIM2 recognizes cytosolic dsDNA from vacuolar bacteria that escape to the cytosol and recruits ASC for caspase 1 activation [75]. Necrotic cell DNA can also be detected by AIM2 [76].

Caspase 11, the intracellular sensor of LPS and a noncanonical inflammasome, induces pyroptosis without additional proteins/adaptors and does not process IL1*β* and IL18 directly [61, 62]. Mouse caspase 1 and caspase 11 cleave the protein gasdermin D (GSDMD) to induce pyroptosis [77, 78]. The N-terminal fragment of GSDMD binds to membrane lipids, then oligomerizes to form pores resulting in cell lysis [79]. Caspase 4 and caspase 5 are human orthologs of murine caspase 11 and detect cytosolic LPS [80–83]. The transcription of caspase 4 and caspase 5 is upregulated by LPS or IFN*γ* [84]. Caspase 11-deficient mice are highly susceptible to *B*. *pseudomallei* [85] confirming that, at least in mice, caspase 11-mediated pyroptosis is host protective by eliminating the replicative niche for this bacterium.

Most *in vitro* investigations of inflammasome activation using bacterial virulence factors or bacteria mutants lacking these factors were performed with mouse cell lines or bone marrow-derived macrophages. Mouse inflammasomes differ from human inflammasomes, both in ligand specificity and in number and types of inflammasomes. There are three NLRP1 isoforms in mice and only one human NLRP1 with different ligand specificities from mouse NLRP1s [86]. Mouse NLRC4 isoforms can be stimulated by flagellin, T3SS rod, and needle proteins [87]. The only ligand for human NLRC4 is T3SS needle protein [74]. The IFN-induced GTPase proteins, immunity-related GTPase (IRG) and guanylate-binding proteins (GBPs), are critical for modulating macrophage activities including autophagy and inflammasome activation [10]. The human and mouse IFN-induced GTPase proteins are vastly different [88, 89]. Mice have more IRGs and GBPs, each with more diverse functions when compared to human IFN-induced GTPase proteins. These differences create some confusion regarding NLR activation by different PAMPs and their regulation by IFNs in human and mouse macrophages.

Murine NLRC4 does not bind to flagellin or T3SS rod and needle proteins directly, rather it requires NAIPs for binding, which then recruit NLRC4 to assemble a canonical caspase 1-dependent inflammasome in the cytoplasm [74, 87, 90]. *B*. *pseudomallei* flagellin binds to NAIP5 and activates NLRC4 in mouse macrophages. In a murine model of respiratory melioidosis, TLR5 and NLRC4 each contributed equally to survival as single-knockout mice were as susceptible as animals deficient in both TLR5 and NLRC4 [91]. Thus, TLR5 and NLRC4 have nonredundant functions in pulmonary melioidosis, although they both bind to flagellin [91]. Interestingly, deficiency of caspase 1 and caspase 11 in this mouse model leads to more severe pulmonary inflammation than deficiency of NLRC4, which demonstrates cooperation between the different types of inflammasomes.

The flagellin of *L*. *pneumophila* binds to NAIP5 and oligomerizes with NLRC4 to form an inflammasome in mouse macrophages [73]. NLRC4 interacts with caspase 1 directly through its respective CARD without recruitment of ASC. NLRC4 and NAIP5 also stimulate autophagosome turnover and coordinate pyroptosis with autophagy to prevent excessive cell death in *L*. *pneumophila*-infected macrophages [92]. It appears that in unstimulated mouse macrophages from resistant C57B6 mice, NLRC4 associates with autophagic protein beclin 1/Atg6. Upon *Legionella* infection, PAMPs binding to NLRC4 release beclin 1 to induce autophagy. This autophagic response protects macrophages from caspase 1-mediated pyroptosis. Thus, unlike humans, C57B6 mice resist *L*. *pneumophila* by increasing autophagy when inflammasome NLCR4 is activated by flagellin. Considering that autophagy also controls IL1*β* by targeting IL1*β* for degradation [93], the upregulation of autophagy after NLRC4 activation by *Legionella* PAMPs is likely a host feedback mechanism to control bacterial replication. *Mtb* only activates the NLRP3 inflammasome, likely by potassium depletion after phagocytosis [94]. The production of IL1*β* by inflammasomes during infection with *Mtb* is critical for a protective host immune response. In contrast, IFN*β* induced after *Mtb* infection can suppress NLRP3 inflammasome activation. Inflammasome regulation by both IFN*β* and IL1*β* illustrates the opposing role of cytokines in modulating inflammation and cell death.

The T7SS effector ESX-1 of *Mtb* damages the phagosomal membrane and releases phagosomal mycobacterial products to the cytosol [95]. One such product is bacterial DNA, which is detected by cytosolic DNA sensors and induces IFNI [96]. The induction of IFNI by *Mtb* is associated with mycobacterial virulence and macrophage necrosis [97]. Another sensor of cytosolic bacterial DNA is cyclic GMP-AMP synthase (cGAS). cGAS binds to endoplasmic reticulum-associated adaptor protein STING (stimulator of IFN genes) which then recruits other IFN-inducing proteins including IRF3 [98]. The cGAS/STING pathway induces IFN, cytokines, and chemokines. *Legionella* and *Mtb* DNA can be detected by cGAS/STING, inducing IFNs which can indirectly modulate inflammasomes [98, 99].

## 5. Phagocyte Cell Death Modes Determine Pathogenesis

Induction of cell death is an effective strategy used by macrophages to clear intracellular pathogens by expelling bacteria from their replicative niche [4, 5]. Paradoxically, successful intracellular bacteria modulate different forms of cell death in phagocytes: apoptosis, pyroptosis, and necrosis/necroptosis to evade host defense [100, 101]. The mode of cell death in phagocytes is highly dependent on the bacterial load and secreted bacterial cytotoxins. The Cif (cycle inhibiting factor) homolog in BP (CHBP) effector induces apoptosis in mouse BMDM as it deamidates host ubiquitin (Ub) and an Ub-like protein [102].

Apoptosis is an active programmed process of packaging cell contents for elimination without inducing inflammation and is dependent on sequential proteolytic activation of caspases [103]. Apoptosis has homeostatic functions during development, infection, and the recovery phase of infection, as this form of cell death is noninflammatory [104, 105]. An apoptotic cell packages cellular contents into membrane-bound apoptotic bodies which are cleared later by another phagocyte in a process termed efferocytosis [105]. Apoptotic cells display many recognition receptors for their removal by phagocytes [106]. However, the uptake of apoptotic cells leads to a reduction of antimicrobial activities in the efferocytosing cell [105]. Uncleared apoptotic cells usually become necrotic and release cellular DAMPs to promote tissue injury. In the context of intracellular infection, excess apoptosis may also be detrimental as efferocytosing phagocytes are less antimicrobial and contribute to bacterial expansion and persistence.

The extrinsic or intrinsic pathways of apoptosis are mediated by ligands binding to the TNF receptor superfamily or by mitochondrial damage, respectively [103]. Extrinsic apoptosis is dependent on caspase 8 activation whereas intrinsic apoptosis is dependent on caspase 9. Both pathways are dependent on caspase 3 and caspase 7 as the executioners of apoptosis. The TNF receptor family includes TNF*α* receptor (TNFR1) and death receptors and activates the extrinsic pathway of apoptosis upon binding to their respective ligands. Induction of apoptosis of infected cells likely includes both the extrinsic and intrinsic pathways. TNF*α* produced by PAMP activation of TLRs in infected cells triggers the extrinsic pathway. Simultaneously, the intrinsic pathway of apoptosis is activated by mitochondrial ROS and damage induced by intracellular pathogens.

TNFR1 contains a death domain (DD) and can trigger apoptosis, necroptosis, and NF*κ*B-activated prosurvival signals depending on the different signaling complexes formed after binding to TNF*α* [107]. The primary function of TNFR1 signaling is proinflammatory gene induction, with the cell death pathway as a backup response when infection is unresolved [39]. The TNFR1 signaling complex for NF*κ*B activation is highly dependent on the presence of cellular inhibitors of apoptosis proteins (cIAP1 and cIAP2) [107, 108]. Other DD-containing death receptors include the FAS (CD95) and TNF-related apoptosis-inducing ligand (TRAIL) receptors which are also induced by intracellular bacteria. Ligand binding to FAS and TRAIL receptors induces either apoptosis or necroptosis depending on the activation status of the receptor-interacting serine/threonine-protein kinase 3 (RIPK3) [107]. The death-inducing signaling complexes for apoptosis and necroptosis formed by TNFR1, FAS, and TRAIL receptors are similar, initiating necroptosis when RIPK3 is activated by phosphorylation.

Necrosis is a form of lytic cell death used by some bacteria to disseminate and evade host immune responses. *Mtb* infects macrophages and induces necrosis to avoid the intracellular immune response in macrophages and to disseminate [109]. Virulent *Mtb* strains induce macrophage necrosis and IFNI, whereas avirulent strains do not [99, 109]. *Mtb* replicates in necrotic macrophages, underscoring its persistence even after macrophage demise. Necroptosis is programed necrosis that is induced when caspase 8 activity is blocked [107]. Necroptosis occurs when the homologous serine/threonine kinases, RIPK1 and RIPK3, interact through their RHIM (RIP homotypic interaction motif) resulting in RIPK3 phosphorylation and activation. RIPK3 then phosphorylates and activates the pseudokinase MLKL (mixed lineage kinase domain-like). Activated MLKL induces pore formation in the plasma membrane and cell leakage of DAMPs. A number of recent studies reveal that the apoptotic caspase 8 has many other activities, including suppression of necroptosis by cleavage of RIPK1, RIPK3, and caspase 1; proteolytic processing of IL1*β* to its mature active form; priming NLRP3; and regulation of cytokine transcriptional responses [110, 111]. The crossover inflammatory activities of caspase 8 indicate that caspase 8 also acts as an inflammasome depending on its catalytic action on caspase 1, IL1*β*, RIPK1, and RIPK3, as well as the absence or depletion of caspase inhibitors.

Canonical inflammasomes NLRP3, NLRC4, and AIM2 recruit caspase 1 through the CARD of NLR or recruited ASC adaptor. Autoproteolytic activity of caspase 1 results in cleavage of pro-IL1*β*, pro-IL18, GSDMD, and pyroptosis. Human macrophage THP-1 cell line and mouse peritoneal macrophages infected with *B*. *pseudomallei* succumb to caspase 1-dependent pyroptosis, presumably via NLRP3 activation [112]. *In vitro*, peritoneal macrophages from caspase 1-deficient mice are resistant to *B*. *pseudomallei*-induced cytotoxicity [112]. Caspase 11 also promotes pyroptosis but with inefficient cytokine production. Caspase 1 and caspase 11 knockout mice are highly susceptible to *B*. *pseudomallei*, and both types of mice have higher bacterial burdens [85, 113]. IL1*β* and the IFN*γ*-inducing factor IL18 released by canonical inflammasomes are inflammatory and host protective, as IL1*β* recruits neutrophils to sites of infection and IFN*γ* activates phagocyte microbicidal activities. In murine melioidosis, multiple T3SS effectors activate NLRC4 to induce caspase 1 and pyroptosis in BMDM [114]. *Legionella* also activates NLRC4, and in resistant C57B6 macrophages, NLRC4 inflammasome activation induces autophagy to dampen inflammation [92]. Neutrophils are unable to undergo pyroptosis and continue to produce IL1*β* after bacterial infection [115]. Unlike most other bacteria, *B*. *pseudomallei* replicates in both macrophages and neutrophils [8, 29]. Thus, neutrophils harboring *B*. *pseudomallei* can be taken up by another phagocyte, either a macrophage or a neutrophil, promoting infection by cell-to-cell spread [116]. Pyroptosis contributes to pathogen clearance in *Legionella* infection by removing the macrophage, their only replicative niche. In contrast, pyroptosis of *B*. *pseudomallei*-infected macrophages may not be a beneficial host response as DAMPs released from cell lysis promote tissue necrosis and lower the infection barrier. The continued IL1*β* release by inflammasomes fuels neutrophil recruitment, which is detrimental, since *B*. *pseudomallei* replicates in neutrophils. Dampening pyroptosis and necroptosis is central to control tissue injury and host survival.

## 6. Cross-Regulation of Inflammasome, Autophagy, and Cell Death in Bacterial Infection

Inflammasome activation by cytosolic PAMPs and DAMPs promotes proinflammatory cytokine production and different types of cell death. Macrophage apoptosis limits cells from further cytokine production and restricts bacteria that cannot replicate in other cell types. Macrophage pyroptosis induced by inflammasomes after detecting cytosolic bacteria expels intracellular bacteria from their replicative niche but also releases proinflammatory IL1*β* and DAMPs. IL1*β* is generally host protective against bacteria such as *Mtb* and *Legionella* but is inflammatory nonetheless. IL1*β* recruitment of neutrophils is damaging in *B*. *pseudomallei* infection, since this bacterium replicates in neutrophils, evades autophagy, and activates inflammasomes. C57B6 macrophages resist *Legionella* infection by increasing autophagy to eliminate bacteria [92]. Cytokines IL1*β*, TNF*α*, and IFNs induced by TLRs and inflammasomes via pathogens, PAMPs, and DAMPs modulate inflammasomes, autophagy, and cell death depending on the timing of cytokine induction and cellular context. TNF*α* can induce different types of cell death depending on the formation of death complexes with caspase 8 and cIAPs as critical modulators of necroptosis. IFNI plays a critical role in cell death as it synergizes with TNF*α* to induce necroptosis. Basal IFNI is protective against certain pathogens by sustaining key intermediates of signaling molecules that have antimicrobial activities [10, 88, 117]. IFN*β* signaling induces caspase 11 and AIM2 expression, leading to enhanced cytosolic sensing of bacterial contamination in the cytosol [10, 118]. In addition, IFN-induced IRGs and GBPs play pivotal roles in antimicrobial defense against intracellular bacteria, especially those that reside in vacuoles. GBPs recognize specific host lipid molecules on pathogen-containing vacuoles and mark them for disruption or delivery to lysosomes. Thus, IFNI promotes clearance of *Legionella* as it disrupts vacuolar compartments where the bacterium localizes. The coordination of pyroptosis and enhanced autophagy by inflammasome activation in *Legionella* infection results in bacterial clearance and survival of C57/B6 mice [92]. Cytosolic ubiquitylated bacteria are also detected by GBPs which mark them for autophagic degradation. Host-intrinsic IFN-sustained GBPs promote NLRP3, caspase 1, and caspase 11 activation. IFNI, IL1*β*, and TNF*α* fine-tune inflammasomes, autophagy, and caspase 8-mediated cell death modes to control many aspects of bacterial infections. A summary of the interactions of autophagy, inflammasomes, and host cell death modes with *B*. *pseudomallei*, *Mtb*, and *Legionella* is presented in Table 1.

## 7. Conclusions and Perspectives

Autophagy, inflammasome, and cell death are cellular processes fundamental to innate host defense against pathogens. Initiation of autophagy upon phagocytosis ensures pathogen capture and elimination. Inflammation via inflammasome activation after sensing pathogens in the cytosol clearly guards host cells against microbial contamination in the cytosol. TLRs, NLRs, AIM2, and noncanonical inflammasomes caspase 11 and caspase 8 cooperate to ensure a robust immune response by induction of inflammatory mediators to recruit and activate phagocytes to kill pathogens. Pyroptosis of macrophages, induced by caspase 11 and NLRs, expels cytosolic bacteria from their replicative niche. This form of inflammatory cell death has detrimental consequences as macrophage death decreases the very cell that can kill microbes and releases DAMPs that further stimulate inflammation and prevent resolution of disease and pathogen clearance. In *B*. *pseudomallei* infection, inflammasome activation, pyroptosis, and necroptosis appear to be detrimental due to excessive cell death and collateral damage in vital organs. A better understanding of the cross-regulation of these processes of innate host defense against intracellular bacteria might facilitate target identification for host-directed therapeutics against pathogens.

## Figures and Tables

**Table 1:** Interplay of *Burkholderia pseudomallei* (*Bp*), *Mycobacterium tuberculosis* (*Mtb*), and *Legionella pneumophila* (*Lp*) with autophagy, inflammasomes, and host cell death modes.

	Autophagy	Inflammasomes	Cell death
*Bp*	T3SS effectors aid bacterial escape to the cytosol [13, 14]. Bacteria evade autophagy [27].	LPS is a weak agonist of TLR4 [54]. Cytosolic LPS activates caspase 11 [85]. Flagellin activates TLR5 [63] and NAIP5/NLRC4 [90, 114].	Caspase 1 and caspase 11 mediate pyroptosis [85, 112, 113]. Caspase 1- and caspase 11-knockout mice are highly susceptible to pyroptosis [85, 113]. T3SS BsaK induces pyroptosis [114].

*Mtb*	Autophagy restricts *Mtb* growth [30]. SapM prevents phagosome maturation [17]. Viable *Mtb* disrupts phagolysosome maturation [31].	ESX-1 induces lysosomal release of IL1*β* and IL18 [95]. ESAT-6 activates NLRP3 and ruptures phagosomes [94]. *Mtb* DNA released by ESX-1 activates cGAS/STING/IFNI [96, 99].	Virulent *Mtb* promotes macrophage necrosis via ESX-1 [95, 109]. Phagosome rupture induces necrosis [97]. Virulent *Mtb* induces IFNI to suppress inflammasomes [41] and promotes necrosis [97].

*Lp*	RavZ inhibits autophagy [32]. T4SS Icm/Dot promotes specialized vacuoles for bacterial replication [16, 23].	Lipopeptides activate TLR2 and limit bacterial replication [57, 58]. Flagellin activates NAIP5/NLRC4 [73] and enhances autophagy [92]. cGAS/STING/IFNI activation targets vacuoles to eliminate bacteria [118].	NAIP5/NLRC4 activation promotes pyroptosis [73]. Coordination of pyroptosis and autophagy by inflammasomes prevents excessive cell death in C57/B6 mice [92].
